# Effects of Chlorogenic Acid on Deoxynivalenol (DON)-Induced Ferroptosis in Porcine Alveolar Macrophages

**DOI:** 10.3390/toxins18060260

**Published:** 2026-06-09

**Authors:** Jinglan Zhang, Xinuo Lai, Zhiwei Na, Junliang Deng, Zhihua Ren, Tong Fu

**Affiliations:** 1 College of Veterinary Medicine, Henan Agricultural University, Zhengzhou 450046, China; 2 College of Veterinary Medicine, Sichuan Agricultural University, Chengdu 610000, China; 3 Department of Animal Science and Technology, Heilongjiang Agricultural Economy Vocational College, Mudanjiang 157041, China; 4 Ministry of Education Key Laboratory for Animal Pathogens and Biosafety, Zhengzhou 450046, China; 5 College of Animal Science and Technology, Henan Agricultural University, Zhengzhou 450046, China

**Keywords:** chlorogenic acid (CGA), deoxynivalenol (DON), ferroptosis, oxidized lipid metabolism

## Abstract

Deoxynivalenol (DON) is a mycotoxin commonly found in food crops and animal feed worldwide. Its pronounced toxicity in pigs poses a serious risk to the swine industry and to human health. This study focused on two central features of ferroptosis—iron metabolism and lipid peroxidation—and examined how chlorogenic acid (CGA) affects DON-induced ferroptosis in porcine alveolar macrophages (PAMs) via cell-based assays and oxidative lipid metabolomics. These findings show that DON disrupts intracellular iron homeostasis by altering iron-handling proteins (upregulating TFR1 and DMT1 and downregulating FPN1), which may lead to iron overload. Concurrently, DON impairs the GPX4 antioxidant axis (downregulating GPX4, SLC3A2, SLC7A11, and GCLC) and increases ROS, and exposure led to a significant increase in numerous oxidized lipid metabolites, consistent with elevated lipid peroxidation, culminating in ferroptosis in PAMs. CGA mitigates these effects by restoring iron homeostasis and reestablishing GPX4 axis function, thereby reducing oxidative stress. Moreover, CGA suppresses lipid peroxidation pathways, notably linoleic acid oxidation metabolism. In conclusion, CGA protects PAMs and mitigates the proferroptotic effects of DON.

## 1. Introduction

Worldwide, various feeds and raw materials are heavily contaminated by mycotoxins. Among these compounds, deoxynivalenol (DON) is the most prevalent, showing the highest detection rate and the greatest rate of exceedance of standards in recent years [1,2,3,4]. DON can induce an overload of intracellular lipid peroxidation while disrupting iron metabolism homeostasis, ultimately resulting in ferroptosis within cells [5,6]. The pathological conditions characterized by a production rate of reactive oxygen species (ROS) that surpasses the scavenging capacity of the endogenous antioxidant system, leading to oxidative damage to biomolecules such as DNA and proteins, are referred to as oxidative stress [7]. Iron overload and oxidative stress during ferroptosis impair immune cell polarization, causing dysfunction and, in many cases, cell death across diverse diseases. Ferroptosis also amplifies and propagates oxidative stress, which culminates in immunosuppression [8]. DON induces ferroptosis in cells, leading to extensive cytotoxic effects in the organism, with immune cells being particularly vulnerable. Notably, pigs exhibit increased susceptibility to DON, thereby presenting a significant risk to China’s swine farming sector. Immune impairment increases the susceptibility of animals to infectious disease and reduces vaccine efficacy; thus, mycotoxicosis is often called an “underlying disease.” Porcine alveolar macrophages (PAMs) are key components of the innate immune system and serve as primary targets for viruses such as porcine reproductive and respiratory syndrome virus, porcine circovirus, and African swine fever virus [9,10,11]. Previous studies have indicated that ferroptosis contributes to the cytotoxic mechanisms of certain mycotoxins [12]. However, no studies have examined whether DON induces ferroptosis in PAMs as a mechanism of its cytotoxicity.

Various approaches have been explored to mitigate deoxynivalenol (DON) toxicity, including composite adsorbents (e.g., montmorillonite powder and chitosan), chemical agents (e.g., schisandrin A), probiotics (e.g., *Saccharomyces cerevisiae*), and advanced degradation technologies such as photocatalysis [13,14,15,16,17]. However, their high cost, residual toxicity, nutrient loss, and reliance on complex equipment have limited their practical application. Currently, the addition of plant-derived monomeric mycotoxin antagonists is an important strategy for preventing and controlling mycotoxin contamination. Chlorogenic acid (CGA) is a polyphenolic compound widely present in plants such as *Eucommia ulmoides*, *Lonicera japonica*, *Stevia rebaudiana*, *Taraxacum mongolicum*, and coffee. *Lonicera japonica* and *Eucommia ulmoides* leaves are the most common extraction sources, since they contain high concentrations of CGA, and their extraction methods are well established. CGA has diverse functions, such as broad-spectrum antibacterial activity, mold growth inhibition, antioxidant properties, a reduction in ROS accumulation, and immunomodulation [18,19]. Previous research by our group and existing studies have shown that CGA compounds can modulate key proteins associated with ferroptosis and partially suppress its onset [20]. Nevertheless, there is a lack of research on the influence of CGA on DON-induced ferroptosis in PAMs. Investigating this influence and its underlying mechanisms offers insights for mitigating mycotoxin pollution in swine production and is crucial for the sustainable growth of the agricultural breeding sector.

## 2. Results

### 2.1. Optimization of the Concentrations of DON and CGA

**Figure 1:**
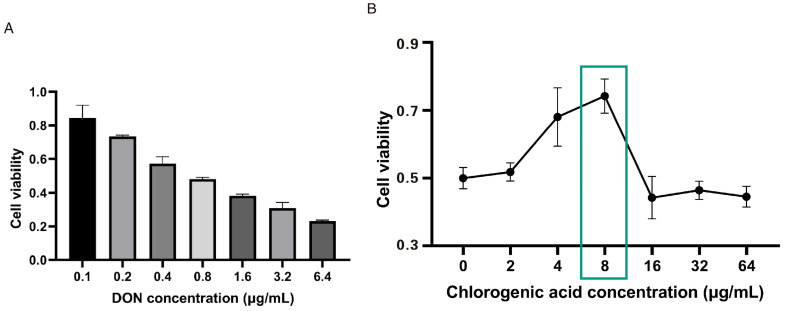
Determination of the experimental concentrations of DON and CGA. (**A**) Effects of varying DON concentrations on the relative viability of PAMs (*n* = 3). (**B**) Effects of different CGA concentrations on the relative viability of PAMs exposed to DON (*n* = 3).

Cell viability was measured by the CCK-8 assay, and the results are shown in Figure 1A,B. PAM survival progressively decreased with increasing DON concentration. The CC_50_ of DON for PAMs was 2.84 μM (0.84 μg/mL). At this DON concentration, the PAM viability curve reached its maximum at 8 μg/mL CGA, where PAM viability was significantly improved (*p* < 0.01). This concentration provided substantial protection to PAMs, so 8 μg/mL CGA was applied for subsequent experiments.

### 2.2. Effects of CGA on the Expression of Key Proteins Related to Ferroptosis in PAMs Exposed to DON

#### 2.2.1. Effects of CGA on the Expression of Iron Metabolism Proteins in PAMs Exposed to DON

**Figure 2:**
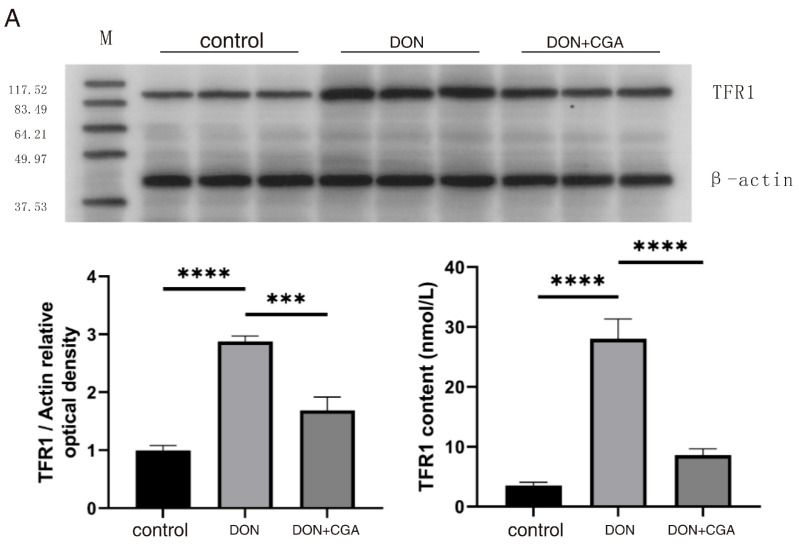

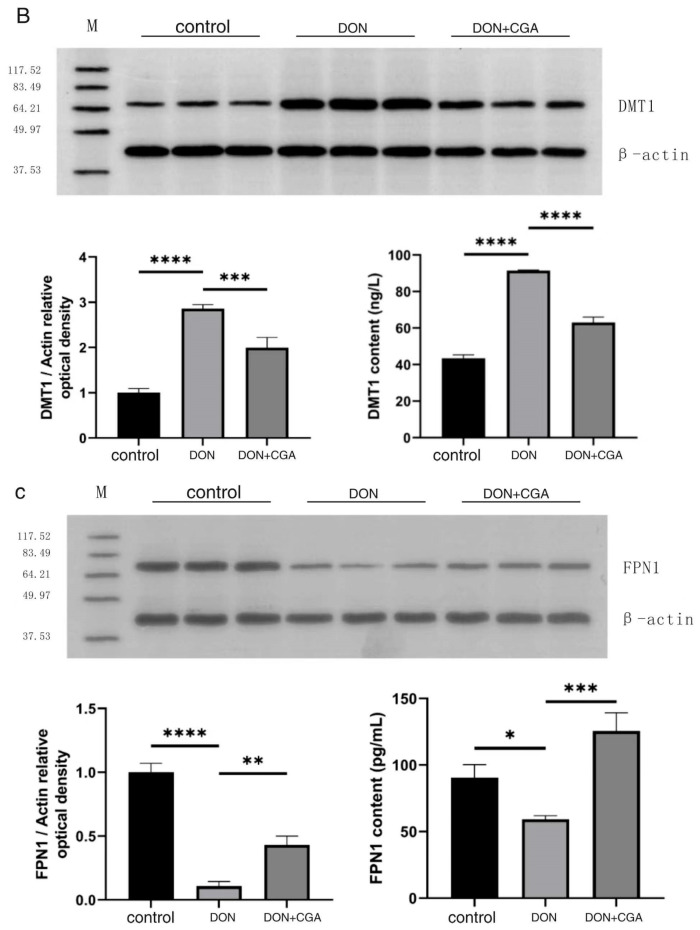
Effects of CGA on the expression of iron metabolism proteins in PAMs exposed to DON. (**A**) Intracellular TFR1 expression in PAMs exposed to DON following CGA treatment (n = 3). (**B**) DMT1 expression in PAMs exposed to DON following CGA treatment (n = 3). (**C**) FPN1 expression in PAMs exposed to DON following CGA treatment (n = 3). Note: The Western blot images with two bands represent the target protein and the internal control protein β-actin. Significance is interpreted as follows: significant (* *p* < 0.05, ** *p* < 0.01, *** *p* < 0.001, **** *p* < 0.0001), unmarked or “ns” indicates no significant difference (*p* > 0.05). M indicates protein marker.

The Western blot and ELISA results are shown in Figure 2A,B. DON significantly upregulated intracellular TFR1 (90 kDa) and DMT1 (65 kDa) in PAMs (*p* < 0.0001). CGA significantly antagonized this effect, significantly reducing TFR1 and DMT1 expression (*p* < 0.001). As shown in Figure 2C, DON significantly downregulated intracellular FPN1 (62–70 kDa) in PAMs (*p* < 0.05). CGA counteracted this suppression and significantly increased FPN1 expression (*p* < 0.01).

#### 2.2.2. Effects of CGA on the Protein Expression of the GPX4-Axis Antioxidant System and the ROS Content in PAMs Exposed to DON

**Figure 3:**
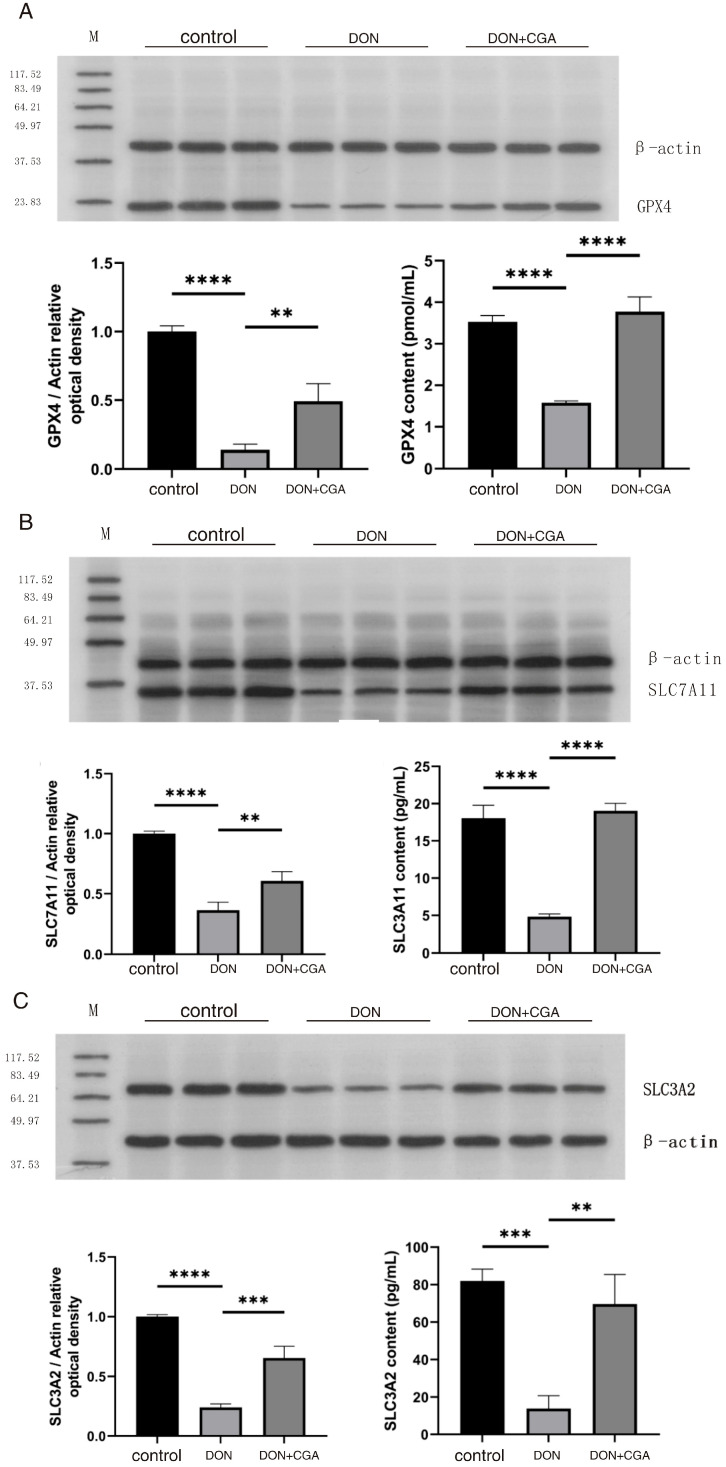

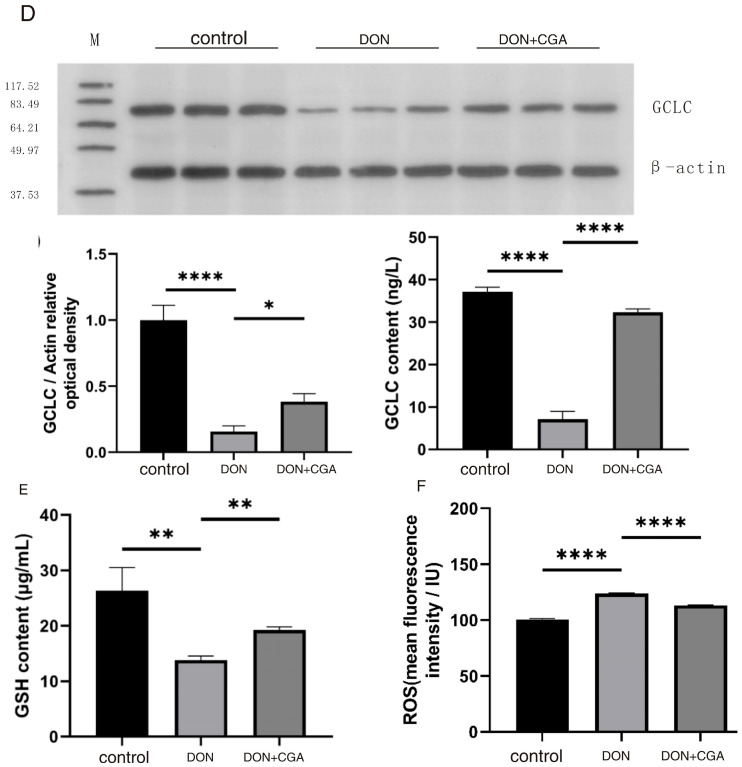
Effects of CGA on GPX4-axis-related antioxidant protein expression and on GSH and ROS levels in PAMs exposed to DON. (**A**–**D**) Effects of CGA on GPX4, SLC7A11, SLC3A2, and GCLC expression in PAMs exposed to DON (n = 3). (**E**,**F**) Effects of CGA on the GSH and ROS contents in PAMs exposed to DON (n = 3). Significance is interpreted as follows: significant (* *p* < 0.05, ** *p* < 0.01, *** *p* < 0.001, **** *p* < 0.0001), unmarked or “ns” indicates no significant difference (*p* > 0.05). M indicates protein marker.

Figure 3A–D present the detection results for GPX4 (20–23 kDa), SLC7A11 (35–40 kDa), SLC3A2 (67 kDa), and GCLC (72 kDa). Both Western blotting and ELISA revealed that, relative to the control group, the DON group showed a significant decrease in the expression of these proteins in PAMs (*p* < 0.0001), indicating that DON toxicity suppresses their expression. Compared with the DON group, the protein levels in the DON + CGA group were significantly increased (*p* < 0.05), indicating that CGA antagonizes the DON-induced inhibition of these proteins in PAMs.

The results of the GSH content measurements are shown in Figure 3E. DON caused a significant decrease in the GSH level (*p* < 0.01), indicating the inhibition of GSH synthesis in PAMs. CGA produced a significant increase in GSH (*p* < 0.01), mitigating the inhibitory effect of DON.

The ROS measurements are presented in Figure 3F. Compared with those in the control group, DON treatment increased intracellular ROS levels. CGA significantly reduced the ROS content (*p* < 0.0001), mitigating the effect of DON, and decreased intracellular ROS accumulation.

### 2.3. Effects of CGA on Oxidative Lipid Metabolism in PAMs Exposed to DON

#### 2.3.1. Screening of Differentially Abundant Metabolites via Oxidative Lipid Metabolomics

**Figure 4:**
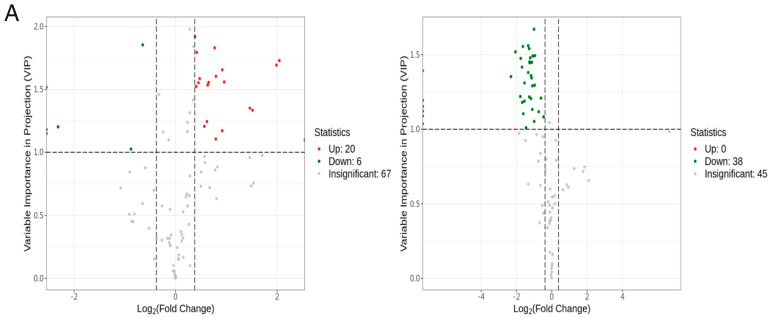

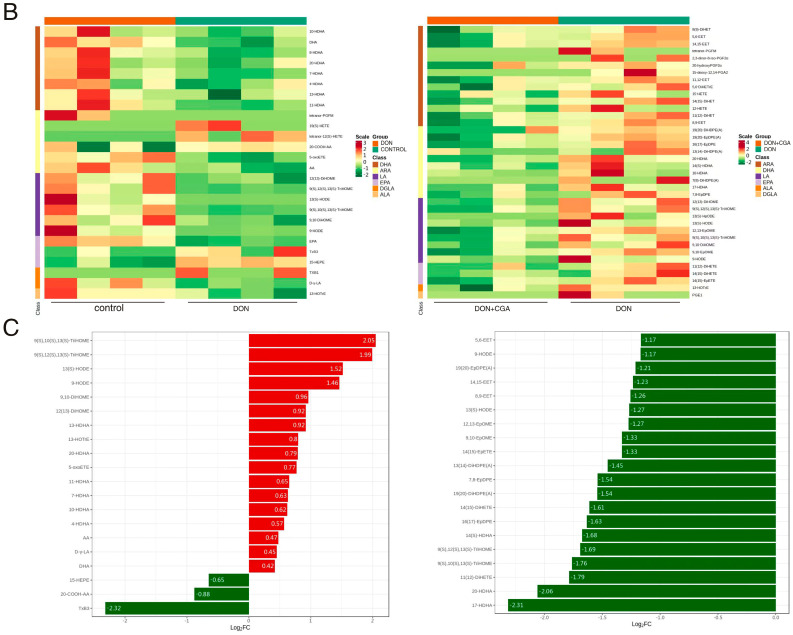
Differentially abundant metabolites were screened via oxidative lipid metabolomics. (**A**) Volcano plots of differentially abundant metabolites: the left panel compares the DON group with the control group, and the right panel compares the DON+CGA group with the DON group. Each point represents a metabolite; green points indicate downregulated differentially abundant metabolites, red points indicate upregulated differentially abundant metabolites, and gray points indicate detected metabolites that are not significantly different. The horizontal axis shows the logarithm (log_2_FC) of the fold change between the two groups; larger absolute values indicate greater differences in metabolite abundance. Under the dual screening criteria of VIP and FC, the vertical axis displays the VIP value; higher VIP values indicate more significant and more reliable differentially abundant metabolites. (**B**) Heatmap of differentially abundant metabolites. The left panel compares the DON group with the control group, and the right panel compares the DON+CGA group with the DON group. The horizontal axis lists the sample names, and the vertical axis lists the metabolites. The scale indicates standardized expression levels (redder colors denote higher expression; greener colors denote lower expression). Group denotes sample grouping, and Class denotes substance classification. (**C**) Fold-change bar chart: The left panel compares the DON group with the control group, and the right panel compares the DON+CGA group with the DON group. The horizontal axis shows the log2FC of differentially abundant metabolites, i.e., the base-2 logarithm of the fold change, and the vertical axis lists the differentially abundant metabolites. Red bars indicate upregulated metabolites, and green bars indicate downregulated metabolites. (DHA: Docosahexaenoic acid; ARA: Arachidonic acid.)

The differentially abundant metabolites between the DON and control groups and between the DON+CGA and DON groups are provided in the Supplementary Materials. As shown in Figure 4A, 20 metabolites were upregulated, and six were downregulated in the DON group relative to the control group, indicating that DON significantly increased the levels of intracellular oxidized lipid metabolites and DON treatment is associated with enhanced lipid peroxidation. In the DON+CGA group, all 38 significantly altered metabolites were downregulated compared with those in the DON group, with some showing large fold changes. These results indicate that CGA reversed the effects of DON on PAM-mediated lipid metabolism at the level of total differentially abundant metabolites and substantially reduced the levels of intracellular oxidized lipid metabolites, thereby inhibiting DON-induced lipid peroxidation.

Differentially abundant metabolites between the two groups were organized by material category and visualized as a heatmap (Figure 4B). The pronounced left–right color contrast in each map indicates significant differences in metabolite abundance between the compared groups. Some metabolites vary among biological replicates within a group because oxidized lipid metabolites are unstable and can degrade under light. Although sample states inevitably differ during experiments and detection, the overall trends remain consistent.

Compared with the control group, the DON group presented the greatest increase in the levels of oxidized lipid metabolites derived from docosahexaenoic acid (DHA) and linoleic acid (LA), with eight and six metabolites, respectively, three of which were arachidonic acid (ARA). Additionally, there was one metabolite each from eicosapentaenoic acid (EPA), dihomo-γ-linolenic acid (DGLA), and alpha-linolenic acid (ALA). Compared with those in the DON group, all of the differentially abundant metabolites in the DON+CGA group were significantly downregulated by CGA. In this comparison, ARA, DHA, and LA accounted for the largest shares, with 14, 10, and nine metabolites, respectively, while the remaining five metabolites were derived from EPA, ALA, and DGLA.

Further analysis of the influence of each control group on the levels of oxidized lipid metabolites is summarized in Figure 4C. The figure displays only the top 20 metabolites with the most significant differences. By fold change, the six most strongly upregulated metabolites under DON treatment were all derived from the LA category, followed by metabolites from the DHA and ARA categories. Notably, DON increases the intracellular levels of the unsaturated fatty acids DHA and AA in PAMs. The metabolites that are differentially regulated by CGA show relatively large fold changes and consist entirely of intermediate or terminal products of oxidized lipid metabolism. Intersecting the differentially abundant metabolites from the two comparisons yielded nine metabolites jointly regulated by DON and CGA, as listed in Table 1. The majority of these shared differentially abundant metabolites belong to the LA category.

#### 2.3.2. KEGG Pathway Enrichment of Differentially Abundant Metabolites

**Figure 5:**
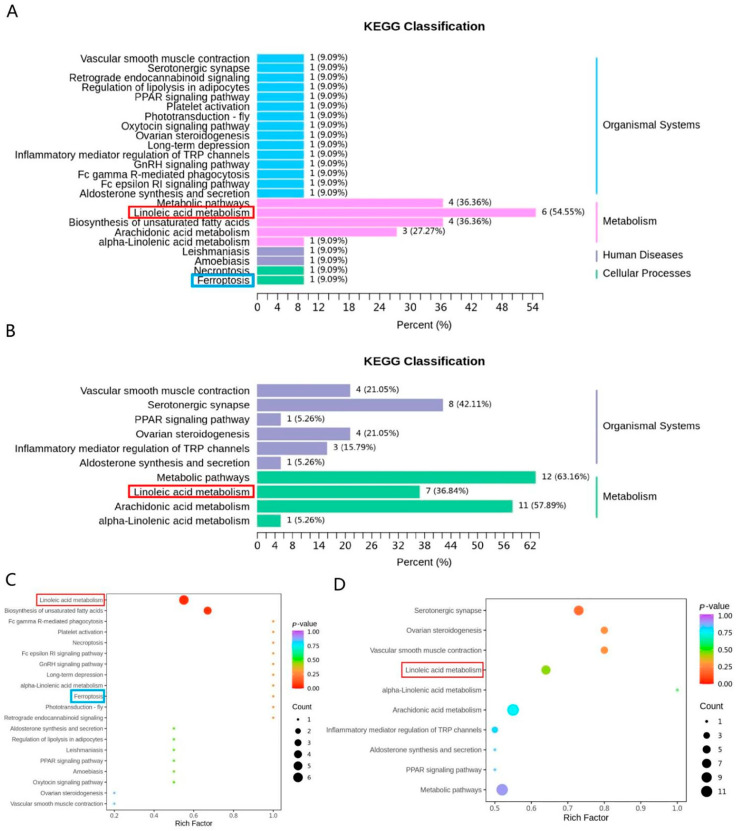
KEGG pathway enrichment of differentially abundant metabolites. (**A**,**B**) The enrichment classification of differentially abundant metabolites in the KEGG pathway. (**A**) Comparison between Group D and Group C. (**B**) Comparison between Group T and Group D. The vertical axis represents the name of the KEGG metabolic pathway, and the number in the figure represents the number of differentially abundant metabolites annotated to the pathway. The value in parentheses represents the ratio of the number of differentially abundant metabolites annotated to the entry to all differentially abundant metabolites annotated to KEGG. (**C**,**D**) Enriched bubble plots of the differentially abundant metabolite KEGG pathways. (**C**) Comparison between group D and group C. (**D**) Comparison between group T and group D. The red box marks the linoleic acid metabolic pathway. The blue box marks the ferroptosis pathway.

Figure 5A–D present the metabolic pathways annotated from the differentially abundant metabolites described above. DON influences these pathways via oxidized lipid metabolites, which span organismal systems, metabolic processes, human diseases, and cellular functions. Metabolism-related pathways were the most affected, with six differentially abundant metabolites mapped to the linoleic acid metabolic pathway. The overall metabolic pathway, the arachidonic acid metabolic pathway, and the biosynthesis pathway of unsaturated fatty acids are also notably impacted. The KEGG enrichment map also revealed that DON strongly affects the linoleic acid metabolic pathway, the alpha-linolenic acid metabolic pathway, and the biosynthesis of unsaturated fatty acids. Notably, only one differentially abundant metabolite is annotated to the ferroptosis pathway, but that metabolite is arachidonic acid (AA), which is upregulated by DON. Its Rich factor value equals 1, indicating that AA is the sole oxidized lipid metabolite originally mapped to the ferroptosis pathway. Thus, the ability of DON to act directly on ferroptosis via this pathway should not be overlooked. The metabolic pathways associated with the differentially abundant metabolites regulated by CGA include both organismal systems and metabolic processes. Among the metabolism-related pathways highlighted in this study, the most prominent was the overall metabolic pathway annotated by 12 differentially abundant metabolites. The linoleic acid and arachidonic acid metabolic pathways are also notable. The KEGG enrichment map emphasized these pathways and further revealed a significant effect of the differentially abundant metabolites in the DON+CGA group on the linolenic acid metabolic pathway.

Overall, the intracellular metabolic pathways in PAMs altered by DON, specifically those influencing the generation of oxidized lipid metabolites, are essentially the same as the pathways altered in PAMs after CGA treatment. This study, therefore, focused on the linoleic acid metabolic pathway to demonstrate that CGA antagonizes DON at the level of oxidized lipid metabolism.

## 3. Discussion

### 3.1. CGA Inhibits DON-Induced Ferroptosis in PAMs by Regulating the Expression of Key Proteins

Ferroptosis is a form of cell death driven by the accumulation of iron ions, so preserving intracellular iron homeostasis is critical to prevent it. In circulation, transferrin (Tf) binds and carries iron as Fe^3+^. Cells primarily take up iron via endocytosis of the Fe^3+^/Tf/transferrin receptor 1 (TFR1) complex. Inside endocytic vesicles, Fe^3+^ is reduced to Fe^2+^, which is then exported into the cytoplasm by divalent metal transporter 1 (DMT1). The imported Fe^2+^ joins the labile iron pool (LIP), where cells either deploy it for metabolic needs or sequester it in storage. Currently, the only confirmed mechanism for cellular iron export is transmembrane transport mediated by ferroportin 1 (FPN1). In contrast, TFR1 and DMT1 mediate the cellular uptake of Fe^2+^, whereas FPN1 facilitates its efflux [21,22]. Ferroptosis is driven by iron-dependent lipid peroxidation; iron ions generate reactive oxygen species (ROS) via the Fenton reaction. Thus, intracellular iron accumulation directly promotes ferroptosis and can disrupt mitochondrial function, further increasing ROS production [23,24].

The results showed that DON significantly upregulated TFR1 and DMT1 expression while downregulating FPN1 expression. This pattern may enhance iron import and reduce iron export, causing intracellular Fe^2+^ accumulation and creating conditions favorable for ferroptosis. In contrast, CGA significantly mitigated the effects of DON on these three proteins, thereby may restoring normal intracellular iron metabolism and preventing DON-induced Fe^2+^ accumulation. CGA also acted as an antioxidant that limits intracellular ROS buildup and mitigates oxidative stress; thereby, CGA can inhibit ferroptosis.

The Xc-/GSH/GPX4 axis is the principal mechanism that prevents ferroptosis in mammals. The light-chain subunit SLC7A11 of system Xc mediates cystine uptake and glutamate export, thereby modulating glutathione (GSH) synthesis. The heavy-chain subunit SLC3A2 maintains system Xc- stability. GSH functions as an essential cofactor for GPX4, which reduces and detoxifies reactive oxygen species (ROS) to suppress ferroptosis. GPX4 prevents lipid peroxidation by reducing lipid peroxides to their corresponding alcohols, thereby suppressing ferroptosis. GSH has antioxidant and anti-inflammatory functions, and its depletion can exacerbate oxidative stress and inflammation. Downregulation of the upstream transporter SLC7A11 impairs cysteine uptake, lowers intracellular cysteine, and depletes GSH biosynthesis. This cascade suppresses GPX4 activity, permits lipid peroxide accumulation, and ultimately induces ferroptosis in cells [25]. Our present experiment corroborated this sequence. DON significantly reduced the protein levels of SLC7A11, SLC3A2, and GPX4, significantly lowered the level of intracellular GSH, and impaired the system Xc-/GSH/GPX4 axis, thereby compromising antioxidant defenses in PAMs. In contrast, CGA mitigated DON-induced inhibition of the system Xc-/GSH/GPX4 axis, partially restoring ROS scavenging and anti-lipid peroxidation capacity, while significantly reducing ROS accumulation and lipid peroxide formation. (Although GSH and ROS levels in the DON + CGA group were significantly improved, they did not return to the levels of the control group, indicating that the protective effect of CGA is partial.) Previous studies have shown that DON suppresses the SLC7A11/GSH/GPX4 axis by downregulating these proteins, leading to the accumulation of ROS [26,27,28]. However, the involvement of CGAs in this mechanism has rarely been reported.

The results showed that DON significantly inhibited GCLC. GCLC catalyzes the conjugation of cysteine and glutamic acid, initiating GSH synthesis and directly linking its activity to the GSH/GPX4 axis. CGA attenuates DON toxicity by increasing GCLC protein expression and preserving the GCLC/GSH/GPX4 pathway. This antioxidant’s ability to mitigate DON toxicity and increase GCLC expression is consistent with previous findings [29].

### 3.2. CGA Inhibits DON-Induced Ferroptosis in PAMs by Reducing Oxidative Lipid Metabolism

Polyunsaturated fatty acids (PUFAs) are prone to oxidation. Under these conditions, they undergo lipid peroxidation, a reaction catalyzed by iron ions that promotes ferroptosis [30]. Ferroptosis features abnormal accumulation of membrane lipid peroxides, leading to membrane rupture, the release of intracellular contents, and the initiation of inflammation. Intracellular iron-storage proteins, such as ferritin, can release iron ions into the extracellular space. Released iron ions can be taken up by neighboring cells, increasing their vulnerability to ferroptosis. Inflammatory mediators in the microenvironment, including tumor necrosis factor (TNF), further amplify this effect. TNF-α activates intracellular signaling pathways and promotes the generation of reactive oxygen species (ROS), thereby creating conditions that favor ferroptosis in adjacent cells. Deoxynivalenol (DON) upregulates polyunsaturated fatty acids (PUFAs) and their corresponding lipid peroxides, indicating that DON treatment is associated with enhanced lipid peroxidation and induces oxidative stress by altering oxidative lipid metabolism. CGA markedly inhibits PUFA accumulation and lipid peroxide formation, counteracting lipid peroxidation and thereby mitigating ferroptosis [5,31,32].

The pathways enriched by the differentially abundant metabolites induced by DON closely match those enriched by the differentially abundant metabolites triggered by CGA, confirming the antagonistic effect of CGA on DON. These pathways involve synthesis routes for polyunsaturated fatty acids, notably the metabolic pathways of LA and ALA, which directly contribute to PUFA production. They also affect the downstream regulation of lipid peroxides and thereby modulate the generation of cellular oxidative stress. The linoleic acid (LA) metabolic pathway is the most representative among these pathways. Analysis of the metabolites regulated in this pathway revealed that those affected by DON and CGA largely overlap [33,34].

As shown in Table 1, multiple linoleic acid (LA) metabolites, such as 9-HODE and 13(S)-HODE, are produced by ALOX15 and cytochrome P450 (CYP) enzymes. The activity of these enzymes directly generates lipid peroxides and consumes intracellular reducing agents (e.g., glutathione, GSH), thereby inhibiting glutathione peroxidase 4 (GPX4)—a central event that triggers ferroptosis. Additionally, 13(S)-HODE inhibits catalase (CAT), leading to hydrogen peroxide (H_2_O_2_) accumulation and further exacerbating oxidative stress. These observations illustrate a robust crosstalk between polyunsaturated fatty acid metabolism and redox balance. Regarding their functional synergy, 9-HODE (generated via the ALOX15 pathway) and 13(S)-HODE (also derived from ALOX15 and known to reduce CAT activity) play dual roles in promoting inflammation and oxidative stress. 9-HODE is a recognized pro-inflammatory mediator that activates G-protein-coupled receptors (e.g., G2A), inducing macrophages to produce chemokines and cytokines. In parallel, 13(S)-HODE directly amplifies oxidative stress by inhibiting CAT, which results in intracellular H_2_O_2_ accumulation. High levels of reactive oxygen species (ROS) not only serve as a key trigger of ferroptosis, but also further amplify inflammatory signals. Persistent elevation of HODEs skews macrophages toward a pro-inflammatory M1 phenotype, thereby impairing their ability to clear pathogens and debris. Moreover, increased ROS levels deplete GSH and suppress GPX4 activity—the core event of ferroptosis—suggesting that these HODEs may directly participate in establishing a ferroptotic microenvironment [35,36,37].

9,10-DiHOME, derived from the cytochrome P450 (CYP) pathway, directly induces oxidative stress, impairs mitochondrial function, and increases lipid peroxidation, thereby providing substrates and conditions conducive to ferroptosis. Among these metabolites, 12(13)-DiHOME plays a more critical role by distinctly influencing T cell differentiation, promoting Th17 cells while inhibiting regulatory T cells (Tregs). Although macrophages are innate immune cells, the cytokine milieu shaped by T cells—such as pro-inflammatory IL-17 from Th17 cells and anti-inflammatory IL-10 from Tregs—reciprocally modulates macrophage function. By tilting the T cell balance, DiHOMEs indirectly drive macrophages toward a dysregulated, pro-inflammatory phenotype. Synergistically, the co-presence of both DiHOMEs not only directly damages macrophages via oxidative stress, but also indirectly disrupts the functional macrophage microenvironment through alterations in adaptive immunity. This creates a positive feedback loop that exacerbates inflammation and tissue damage [38,39,40,41].

9(S),10(S),13(S)-TriHOME and 9(S),12(S),13(S)-TriHOME are both products of the 15-lipoxygenase (15-LOX) pathway. 15-LOX itself catalyzes the peroxidation of phospholipids bearing polyunsaturated fatty acids (PUFAs), thereby directly engaging in the execution of ferroptosis. These TriHOMEs likely represent byproducts or surrogate biomarkers of this process. Their accumulation reflects ongoing localized lipid peroxidation, imposing heightened metabolic stress on macrophages and predisposing them to ferroptosis [42,43,44,45].

Tetranor-PGFM, derived from arachidonic acid (ARA) via the COX-1 pathway, serves as a direct marker of acute inflammation. Its production and the NF-κB inflammatory signaling pathway are mutually reinforcing: inflammatory signals activate COX-1, and the resulting metabolites in turn further activate NF-κB through G protein-coupled receptors (e.g., the TP receptor), establishing a positive feedback loop. This illustrates the crosstalk between fatty acid metabolic pathways and classical inflammatory transcriptional pathways. Tetranor-PGFM (derived from ARA via the COX-1 pathway) is an acute inflammation marker. Prostaglandins originating from COX-1 typically function during the early phase of inflammation or under specific homeostatic conditions; however, their persistent elevation signifies a chronic inflammatory state, which continuously activates macrophages, increases their secretion of reactive oxygen species (ROS) and proteases, and ultimately leads to tissue damage [46].

20-HDHA derived from DHA and 13-HOTrE derived from ALA are precursors or analogs of specialized pro-resolving mediators (SPMs). By binding to specific receptors such as GPR32 and ChemR23, these molecules actively inhibit the NLRP3 inflammasome and promote macrophage polarization toward the M2 phenotype. This illustrates the negative regulatory crosstalk between specific lipid metabolic pathways and innate immune cell polarization pathways. 20-HDHA (derived from DHA via the CYP pathway) may exert inflammation-limiting effects within the macrophage microenvironment, as DHA derivatives often possess pro-resolving functions. Its presence may represent an endogenous compensatory response; however, it is evidently insufficient to counterbalance the effects of other pro-inflammatory metabolites. 13-HOTrE (derived from ALA via the 15-LOX pathway), similar to 20-HDHA, may serve as an endogenous buffering mechanism. Nevertheless, within the overall pro-inflammatory metabolite network, such local anti-inflammatory signals are likely overwhelmed, making it difficult to reverse macrophage dysfunction [47].

Collectively, these metabolites delineate a positive feedback loop. Toxins such as DON or stress signals activate enzymes including CYP, ALOX15, and COX-1, leading to the generation of abundant HODEs, DiHOMEs, and TriHOMEs. These metabolites induce oxidative stress, inhibit catalase (CAT), and promote lipid peroxidation. Consequently, macrophages polarize toward the M1 phenotype, exhibit impaired phagocytic function, and continuously release reactive oxygen species (ROS) and inflammatory cytokines. Meanwhile, DiHOMEs interfere with T cell differentiation and undermine immune regulation. Damage-associated molecular patterns (DAMPs) released from ferroptotic cells, such as lipid peroxides, further activate surrounding immune cells. As a result, the homeostatic balance is disrupted, with the pro-inflammatory/pro-oxidant pathways (LA/ARA metabolism) being substantially upregulated. In contrast, the compensatory capacity of the anti-inflammatory/pro-resolving pathways (DHA/ALA metabolism) proves insufficient to counteract this dysregulation, ultimately leading to macrophage dysfunction and the establishment of a ferroptotic microenvironment. This microenvironment—characterized by heightened oxidative stress, diminished GPX4 activity, sustained inflammatory signaling, and compromised immune regulation—creates a milieu conducive to both the initiation and maintenance of ferroptosis. Therefore, these differential metabolites are not merely biomarkers but also functional effectors [42,43,44,47,48,49,50,51]. To validate their causal roles, a macrophage–fibroblast coculture system could be employed, combined with pharmacological inhibitors targeting 15-LOX (e.g., PD146176) or CYP (e.g., HET0016), to assess whether blocking metabolite production abrogates the ferroptotic phenotype.

Furthermore, DON directly affects the ferroptosis pathway by increasing arachidonic acid (AA) levels. The first step for AA to enter this pathway is the formation of AA-CoA, which is catalyzed by ACSL4. Enzymes such as LPCAT3 then convert AA into PE-AA-O-OH, which participates in Fenton-type reactions to drive oxidative stress and promote ferroptosis. As noted above, DON can influence cellular CoA levels by regulating GCLC expression, suggesting a close link between DON and this pathway. AA can also modulate ferroptosis via alternative routes. Studies have shown that exosomal cir93 enhances AA transport and reacts with taurine to form NATs; this process depletes intracellular AA [40] and reduces ACSL4 and LPCAT3 expression, thereby limiting AA incorporation into the plasma membrane and inhibiting its participation in ferroptosis [52,53].

## 4. Conclusions

This study demonstrates that DON induces ferroptosis in PAMs through a dual mechanism: promoting intracellular Fe^2+^ overload via the dysregulation of TFR1/DMT1/FPN1, and impairing antioxidant capacity via the suppression of the system Xc-/GSH/GPX4 axis and GCLC expression. CGA effectively counteracts DON-induced ferroptosis by restoring iron homeostasis and reconstructing the glutathione-dependent antioxidant system. Lipidomics analysis further reveals that CGA reduces lipid peroxides derived from arachidonic acid, linoleic acid, and DHA, and inhibits the linoleic acid metabolic pathway. Collectively, these findings identify CGA as a ferroptosis inhibitor that targets both iron metabolism and oxidative stress, providing a mechanistic basis for its protective effects against DON-induced cytotoxicity in PAMs. This work also offers new insights into mycotoxin toxicity and establishes a foundation for developing CGA-based interventions against DON-related diseases in livestock.

## 5. Materials and Methods

### 5.1. Cells, Mycotoxin, and CGA

The cells (3D4/21 PAMs) were obtained from Shanghai Meiwan Biotechnology Co., Ltd. (Shanghai, China) and are hereafter referred to uniformly as PAMs. The cells were maintained in RPMI-1640 and subcultured after detachment with 0.25% trypsin.

The DON powder (purity ≥ 99.5%) was acquired from Shanghai Yujing Technology Co., Ltd. (Shanghai, China). Each bottle contained 5 mg of the product with a production batch number of N6000100. The DON powder was subsequently dissolved in dimethyl sulfoxide to create a stock solution with a concentration of 2 mg/mL for storage.

CGA (purity ≥ 99.6%) was procured from Beijing Solaibao Technology Co., Ltd., (Beijing, China) in 5 mg bottles (production batch number: 220501). CGA powder was dissolved in dimethyl sulfoxide to create a 10 mg/mL stock solution for storage.

### 5.2. Experimental Groups

#### 5.2.1. Determination of Cell Viability via the CCK-8 Assay

Cell viability was assessed following the manufacturer’s guidelines with a Cell Counting Kit-8 (Shanghai Biyun Tian, Shanghai, China). The half-maximal inhibitory concentration (IC50) of DON on PAMs and the mitigating effect of CGA on DON-contaminated PAMs were investigated. 

#### 5.2.2. Cell Grouping and Treatment

Following the assessment of DON and CGA in the preceding experiments, the subsequent investigations were categorized into three groups: the normal control group (control group), the group receiving only DON (DON group, DON 2.84 μM), and the treatment group that received both DON and CGA concurrently (DON+CGA group, DON 2.84 μM, CGA 8 μg/mL).

A vehicle control group (0.1% DMSO) was included in all experiments and showed no significant difference from the blank control group.

### 5.3. Detection of GSH via the DTNB Method

The cells were categorized into the previously mentioned control, DON, and DON+CGA groups. Each group underwent treatment for 24 h as described earlier. Three independent biological replicates (n = 3) were established for each group, and cell samples were subsequently collected. Sample processing and procedural steps were meticulously conducted in accordance with the guidelines provided by the Beijing Solaibao Company (Beijing, China) Reduced Glutathione (GSH) Content Detection Kit (BC1175). The standard curve was constructed on the basis of the concentration of the standard solution (x, μg/mL) and the corresponding absorbance (y, △A standard). The absorbance of the test sample (y, △A) was subsequently applied to the standard curve to determine the concentration of GSH in the sample (x, μg/mL). Finally, the GSH content was calculated via the following formula: GSH content (μg/10^6^ cells) = x × V samples ÷ (V samples ÷ total V samples × N) = x ÷ N, where N represents the number of cells measured in millions (10^6^ units).

### 5.4. Detection of ROS via the DCFH-DA Probe Method

The cells were categorized and treated as described in Section 5.3. Cell samples were obtained via an ROS detection kit (CA1410) from Beijing Solabao Company (Beijing, China). Sample preparation and procedural steps were meticulously executed as directed. The approach of introducing probe post-cell collection was adopted. During the resuspension of cells with DCFH-DA diluent, the cell density was maintained at 10^7^ cells/mL. By employing an excitation wavelength of 488 nm and an emission wavelength of 525 nm, the fluorescence intensity in each category was assessed and contrasted.

### 5.5. Detection of Oxidized Lipid Metabolites

The cells were grouped and processed as described in Section 5.3. Following 2–3 washes with PBS, the cells were resuspended and counted. The samples were collected into EP tubes at a concentration of 10^6^ cells per tube, labeled accordingly, and subjected to centrifugation. The supernatant was removed as thoroughly as possible to prepare fresh cell samples. To prevent light exposure and subsequent degradation of oxidized lipid metabolites, the EP tubes were wrapped in tin foil. They were then stored in a freezer box at −80 °C and transported in a dry refrigerator. The analysis of oxidized lipid metabolomics was conducted by Wuhan Maiwei Metabolic Biotechnology Co., Ltd. (Wuhan, China). The data acquisition system comprised ultra-high performance liquid chromatography coupled with tandem mass spectrometry. Chromatographic separation was performed on a Waters ACQUITY UPLC HSS T3 C18 column at 40 °C with a flow rate of 0.4 mL/min. Mobile phase A was 60% acetonitrile (containing 0.002% acetic acid), and mobile phase B was 50% acetonitrile in isopropanol. Mass spectrometry conditions were as follows: electrospray ion source temperature 550 °C, ion spray voltage −4500 V, curtain gas flow 35 psi. In the Q-Trap 6500+, each ion pair was detected using optimized declustering potential and collision energy. Differential metabolites among varieties or tissues were screened using VIP > 1 from the OPLS-DA model, combined with fold change (FC ≥ 1.3 or FC ≤ 0.5).

### 5.6. Western Blot

The cell groups and treatments followed the protocol described in Section 5.3, and the cell samples were collected. Total protein was extracted using a total protein extraction kit (Thermo, Waltham, MA, USA) according to the manufacturer’s instructions, and the protein concentration was determined using the BCA method. Samples from each group were then separated by SDS-PAGE and transferred onto nitrocellulose (NC) membranes. After blocking, the membranes were incubated with primary antibodies against DMT1, TFR1 (ferroptosis-promoting proteins), as well as GPX4, SLC7A11, GCLC, SLC3A2, and FPN1 (ferroptosis-suppressing proteins), followed by chemiluminescent detection using ECL. Western blot analysis was performed as described by Wang M et al. [19]. The expression levels of the ferroptosis-promoting proteins DMT1 and TFR1, as well as the ferroptosis-inhibiting proteins GPX4, SLC7A11, GCLC, SLC3A2, and FPN1, were assessed. The antibody dilution ratio was 1:500, and β-actin was used as the loading control.

### 5.7. ELISA

The cells were categorized into the aforementioned control, DON, and DON+CGA groups. Each group underwent treatment as previously described for a duration of 24 h, with three independent biological replicates (n = 3) established for each group. The culture supernatant was collected in a 2 mL sterile EP tube, centrifuged at 3000 r/min for 20 min, and subsequently transferred to another sterile EP tube. The supernatant was stored at 4 °C and analyzed as soon as possible. The procedure adhered strictly to the instructions provided by the ELISA kits (Shanghai Enzyme-linked Biotechnology Co., Ltd., Shanghai, China) for the detection of the ferroptosis-promoting proteins DMT1 and TFR1, as well as the ferroptosis-inhibiting proteins GPX4, SLC7A11, GCLC, SLC3A2, and FPN1.

### 5.8. Data Processing and Analysis

The data were systematically organized via Excel. One-way ANOVA was performed with SPSS 26.0 software (IBM SPSS, Chicago, IL, USA). Multiple comparisons among groups were conducted via either the least significant difference (LSD) test or Dunnett’s T3 test to evaluate differences in the experimental data. ImageJ software (https://imagej.net/ij/) was used to analyze the gray values of the Western blot bands to calculate the relative protein expression levels. The Western blot, qPCR, and ELISA data were subsequently subjected to one-way analysis of variance (ANOVA) and plotted via GraphPad Prism 9.5.1 software. The results are presented as the means ± standard deviations (means ± SDs). Significance is interpreted as follows: significant (* *p* < 0.05, ** *p*< 0.01, *** *p* < 0.001, **** *p* < 0.0001), unmarked or “ns” indicates no significant difference (*p* > 0.05). M indicates protein marker.

In the KEGG enrichment analysis, the Rich factor (x-axis) is defined as the ratio of the number of differentially abundant metabolites annotated to a given pathway to the total number of metabolites annotated to that pathway. A higher Rich factor indicates a greater degree of enrichment. The *p*-value, calculated by the hypergeometric test, is represented by the color of the dots: a redder color indicates more significant enrichment. The size of each dot reflects the number of differentially abundant metabolites enriched in that pathway. The linoleic acid metabolism pathway is indicated by a red box and the ferroptosis pathway by a blue box.

## Figures and Tables

**Table 1:** Differentially expressed metabolites coregulated by DON and CGA.

Differentially Abundant Metabolites	Substance Categories
9-HODE	LA
9,10-DiHOME	LA
9(S),10(S),13(S)-TriHOME	LA
9(S),12(S),13(S)-TriHOME	LA
13(S)-HODE	LA
12(13)-DiHOME	LA
20-HDHA	DHA
tetranor-PGFM	ARA
13-HOTrE	ALA

## Data Availability

The supporting information and raw data of this article are publicly accessible at https://pan.baidu.com/s/18i4rh0buC3tNJqgTW_RB5Q?pwd=qycc (27 February 2026) and https://doi.org/10.57760/sciencedb.35382.

## Supplementary Materials

The following supporting information can be downloaded at https://www.mdpi.com/article/10.3390/toxins18060260/s1. Figure S1: OPLS-DA validation diagram; Table S1: Chromatographic mobile phase gradient; Table S2: Differential metabolites in the DON group compared with the control group; Table S3: Differential metabolites in the DON+CGA group relative to the DON group.
