# Supplementary material for: Effects of Chlorogenic Acid on Deoxynivalenol (DON)-Induced Ferroptosis in Porcine Alveolar Macrophages
Source: Toxins (Basel). 2026 Jun 9;18(6):260. doi: 10.3390/toxins18060260 (PMC13308313; doi:10.3390/toxins18060260)
Supplement: Supplementary file 1 [file toxins-18-00260-s001.zip › toxins-4343208-supplementary.pdf]

toxins

# Supplementary Materials: Effects of Chlorogenic Acid on Deoxynivalenol (DON)-Induced Ferroptosis in Porcine Alveolar Macrophages

**Jinglan Zhang [1,†], Xinuo Lai [2,†], Zhiwei Na [2,3], Junliang Deng [2], Zhihua Ren [1,2,4,*] and Tong Fu [4,5,*]**

## 1. Materials and Methods

### *1.1. Determination of cell viability by CCK-8 assay*

#### 1.1.1. Resuscitation and culture of passaged cells

(1) Cell passaging

Cells are passaged when the cell confluence is approximately 80% - 90% as observed under an inverted microscope.

① Aspirate and discard the original culture medium in the culture flask.

② Washing: Introduce the PBS washing solution along the wall of the culture flask opposite the side where the cells adhere, and wash the cells three times. Gently agitate the flask to facilitate the natural flow of the liquid over the cell growth area, then aspirate and discard the liquid.

③ Pancreatic enzyme digestion: Introduce 0.25% trypsin to the side opposite the adherent cells, ensuring that the entire cell growth area is covered with the solution. Incubate the cells at 37 °C and 5% CO2 for 3 minutes to facilitate digestion. Subsequently, observe the cells under a microscope. When the cells appear rounded and a few have detached, gently pipette to verify adequate digestion. To terminate the digestion process, add three times the volume of trypsin in complete culture medium. Finally, gently aspirate and agitate to create a cell suspension.

④ Centrifugation: Transfer the prepared cell suspension into a suitable centrifuge tube. Centrifuge at 1500 r/min for 5 minutes, then carefully aspirate the supernatant. The white precipitate at the bottom are the cells.

⑤Resuspend the cell precipitate in complete culture medium, ensuring thorough mixing, and transfer it to new culture flasks. Distribute the suspension evenly into 2-3 flasks as necessary, and subsequently replenish the culture medium in each flask.

⑥ Place it in a constant-temperature incubator and culture it under the conditions of 37 °C and 5% $CO_2$.

（2）Cell cryopreservation

The initial steps of cleaning, digestion, and centrifugation remain consistent with the previously described protocol. Following centrifugation, the supernatant should be discarded. Subsequently, add a serum-free cell cryopreservation solution, mix thoroughly using a pipette, and transfer the mixture to a cell cryopreservation tube. Label the tube appropriately and store it in a -80 °C freezer.

（3）Cell recovery

Remove the cryopreservation tube and thaw it in a 37 °C water bath, ensuring a gradual thawing process. When the contents of the tube reach a mixed state of ice and water, gently mix thoroughly using a pipette and transfer it to a 15 mL centrifuge tube. Add 2-3 mL of complete culture medium and mix thoroughly. Centrifuge at 1500 r/min for 5 minutes, then aspirate and discard the supernatant. Resuspend the cells in complete culture medium, mix well, and transfer the suspension to a culture flask. Adjust the culture medium to an appropriate volume and place the flask in an incubator for cultivation.

 https://doi.org/10.3390/toxins18060260

（4） Cell counting

① The preliminary steps are the same as steps ① - ⑤ of cell passage.

② Transfer a small amount of cell suspension into an EP tube, add PBS for dilution or not according to the situation, and pipette to mix well.

③ Clean the blood cell counter plate and cover slip thoroughly using an alcohol-soaked cotton ball. Position the cover slip at the center of the counter plate, ensuring it overlaps the frosted slope at the edge. Draw 10 μL of the suspension and gradually add it to the groove of the counting plate on the lower side of the cover slip, filling the cover slip completely. Throughout this procedure, it is essential to avoid the formation of bubbles and to prevent the suspension from flowing into the adjacent tank.

④ Under an inverted microscope, the total number of cells (N) in the 16 small cells located at the four corners of the large square was quantified. The calculation formula is as follows: the number of cells in 1 mL of culture medium = N ÷ 4 × 10^4 × dilution factor (cells).

1.1.2. Determination of cell viability by CCK-8 assay

PAMs in the logarithmic growth phase (4 - 5 passages) were enumerated. Following resuspension in maintenance medium, the cells were seeded into 96-well plates based on the experimental groups at a density of $1 \times 10^4$ cells per well. After 24 h of culture, it was observed that the optimal cell confluence per well was 80%. The original medium was aspirated and discarded, and the cells were washed 3 times with PBS. Then, according to the experimental settings, 100 μL of DON at different concentration gradients (0.1 - 6.4 μg/mL) or a mixture of CGA at different concentration gradients (2 - 64 μg/mL) and DON was added to each well. 100 μL of maintenance medium and PBS were added to each well of the normal control group and the blank group respectively. Following a 24 h culture period, cell morphology was observed under a microscope. The original medium was aspirated and the cells were washed with PBS. A mixed solution consisting of 10 μL of CCK-8 reagent and 100 μL of maintenance medium was added to each well. Following a 4 h incubation period, the 96-well plate was placed on a microplate reader hourly to measure the optical density (OD) at a wavelength of 450 nm. Data from the blank group were selected as the reference results. Each treatment group included three replicates.

Calculate the cell survival rate and inhibition rate according to the following formulas:

Survival rate = (A experimental group - A blank group) / (A control group - A blank group) × 100%

*1.2. 4D-DIA proteomic sample detection*

Chromatographic conditions: The chromatographic column utilized was a Waters ACQUITY UPLC HSS T3 C18 column. The column was maintained at a temperature of 40°C, and the flow rate was set at 0.4 mL/min. Mobile phase A consisted of 60% acetonitrile, which contained 0.002% acetic acid, while mobile phase B comprised a 50% acetonitrile-isopropanol solution. The gradient of the chromatographic mobile phase is detailed in Table S1.

**Table S1.** Chromatographic mobile phase gradient.

| Time (min) | A% | B% |
|---|---|---|
| 0 | 99.9 | 0.1 |
| 2 | 70 | 30 |
| 4 | 50 | 50 |
| 5.5 | 1 | 99 |

https://doi.org/10.3390/toxins18060260

| | | |
|---|---|---|
| 7 | 1 | 99 |
| 7.1 | 99.9 | 0.1 |

Mass spectrometry conditions included an electrospray ionization source temperature of 550°C, a mass spectrometry voltage of -4500 V, and a curtain gas flow rate of 35 psi. In the Q-Trap 6500+, each ion pair was analyzed based on optimized declustering voltages and collision energies.

## 2. Results

### *2.1. Determination of the test concentrations of DON and CGA*

In this experiment, the CCK-8 method was employed to assess the cell survival rate of PAMs following treatment with 0-6.4 μg/mL DON for 24 h, thereby evaluating the impact of DON on PAM cell viability. The results are illustrated in Figure 1A of the main text. The figure indicates that as the concentration of DON increases, the survival rate of PAMs progressively declines. Based on these observations, an exponential function was utilized to model the relationship between the survival rate and inhibition rate of PAMs at varying DON concentrations (survival rate + inhibition rate = 1), resulting in two derived formulas. The value y = 0.5, which corresponds to a 50% inhibition rate, was substituted into these formulas for calculation, and the average value was determined. Consequently, the semi-inhibitory concentration (IC50) of DON for PAMs was established at 0.84 μg/mL. This concentration was subsequently selected for further testing.

In this experiment, the CCK-8 method was employed to assess the survival rate of PAMs contaminated with 0.84 μg/mL of DON across a range of CGA concentrations from 0 to 64 μg/mL over a 24 h period. This approach aimed to evaluate the protective effect of CGA on DON-contaminated PAMs. The results are illustrated in Figure 1B of the main text. The figure indicates that the survival rate of DON-contaminated PAMs initially increases and subsequently decreases with rising concentrations of CGA. Notably, the line graph reveals a peak at a concentration of 8 μg/mL, where the viability of PAMs is significantly enhanced ($P < 0.01$). This finding suggests that this specific concentration of CGA mitigates the toxicity of DON, providing substantial protection for PAMs. Consequently, a CGA concentration of 8 μg/mL was selected for subsequent experiments.

### *2.2. Effects of CGA on oxidative lipid metabolism in PAMs exposed to deoxynivalenol (DON)*

#### 2.2.1. OPLS-DA model validation

An in-depth analysis of the metabolomics data was performed using the OPLS-DA model. Score plots for each group were used to visualize metabolic differences between groups. Model prediction performance was assessed using the parameters R2X, R2Y, and Q2. R2X and R2Y indicate the model's explanatory power for the independent variable matrix X and the dependent variable matrix Y, respectively, while Q2 measures prediction accuracy. Values closer to 1 denote greater stability and reliability. Typically, Q2 > 0.5 indicates an effective model, and Q2 > 0.9 denotes excellent performance. As shown in Figure S1, the OPLS-DA model comparing the DON group with the control group yielded Q2 = 0.811 and R2Y = 0.998, and the model comparing the DON+CGA group with the DON group yielded Q2 = 0.614 and R2Y = 0.999. Both sets of results meet the criteria, indicating that the OPLS-DA models for these comparisons are effective. Consequently, the variable importance in projection (VIP) scores derived from the OPLS-DA models are reliable. Differential metabolites were screened by VIP, with VIP > 1 taken to indicate significant differences.

https://doi.org/10.3390/toxins18060260

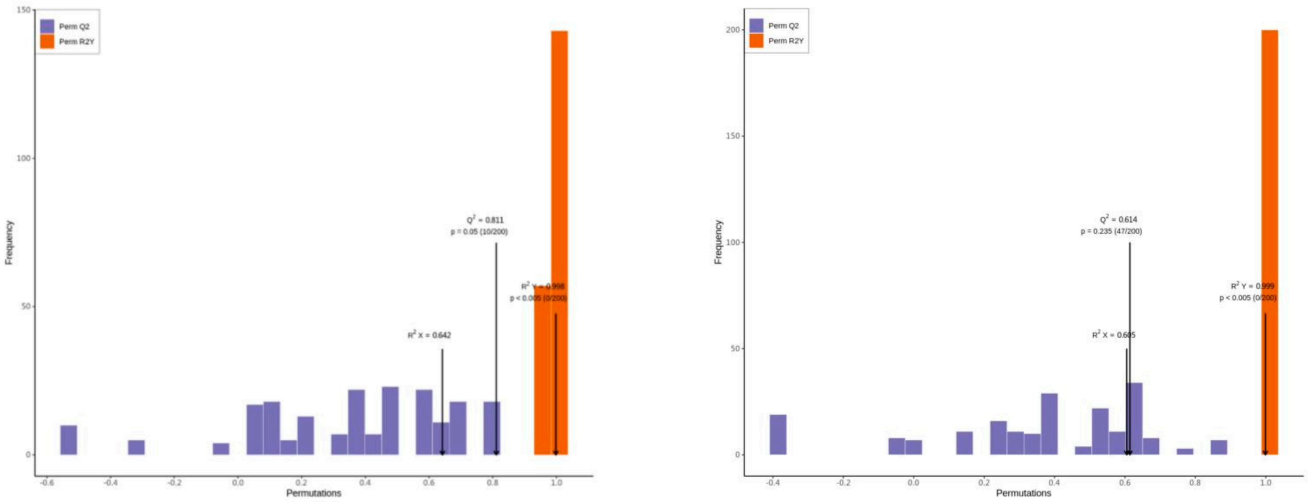


**Figure S1.** OPLS-DA validation diagram.

Note: The left figure shows the OPLS-DA model validation between Group DON and Group control, the right figure shows the OPLS-DA model validation between Group DON+CGA and Group DON. The horizontal axis represents the R2Y and Q2 values, while the vertical axis represents the frequency of model classification performance in 200 times random permutation and combination experiments.

2.2.2. Screening of differential metabolites by oxylipin metabolomics

Table S2 lists the differential metabolites identified by comparing the DON group with the control group, and Table S3 lists those identified by comparing the DON+CGA group with the DON group. As shown in Figure 4A in the main text, the DON group versus control group comparison revealed 20 upregulated and 6 downregulated differential metabolites, indicating that DON markedly increased intracellular oxidized lipid metabolites and promoted lipid peroxidation. In the DON+CGAgroup versus DON group comparison, all 38 significantly altered metabolites were downregulated, with some showing relatively large fold changes. This pattern implies that CGA reversed DON's effect on oxidized lipid metabolism in PAMs, substantially lowering intracellular oxidized lipid metabolites and suppressing lipid peroxidation.

**Table S2.** Differential metabolites in the DON group compared with the control group.

| Substance name | Types of substances | Regulation results |
| --- | --- | --- |
| 13-HOTrE | ALA | up |
| tetranor-PGFM | ARA | up |
| 5-oxoETE | ARA | up |
| AA | ARA | up |
| D-γ-LA | DGLA | up |
| 10-HDHA | DHA | up |
| DHA | DHA | up |
| 8-HDHA | DHA | up |
| 20-HDHA | DHA | up |
| 7-HDHA | DHA | up |
| 4-HDHA | DHA | up |
| 13-HDHA | DHA | up |
| 11-HDHA | DHA | up |

https://doi.org/10.3390/toxins18060260

| | | |
|---|---|---|
| EPA | EPA | up |
| 12(13)-DiHOME | LA | up |
| 9(S),12(S),13(S)-TriHOME | LA | up |
| 13(S)-HODE | LA | up |
| 9(S),10(S),13(S)-TriHOME | LA | up |
| 9,10-DiHOME | LA | up |
| 9-HODE | LA | up |
| 19(S)-HETE | ARA | down |
| tetranor-12(S)-HETE | ARA | down |
| 20-COOH-AA | ARA | down |
| TXB1 | DGLA | down |
| TxB3 | EPA | down |
| 15-HEPE | EPA | down |

**Table S3.** Differential metabolites in the DON+CGA group relative to the DON group.

| Substance name | Types of substances | Regulation results |
|---|---|---|
| 13-HOTrE | ALA | down |
| 8(9)-DiHET | ARA | down |
| 5,6-EET | ARA | down |
| 14,15-EET | ARA | down |
| tetranor-PGFM | ARA | down |
| 2,3-dinor-8-iso-PGF2$\alpha$ | ARA | down |
| 20-hydroxyPGF2$\alpha$ | ARA | down |
| 15-deoxy-12,14-PGA2 | ARA | down |
| 11,12-EET | ARA | down |
| 5,6-DiHETrE | ARA | down |
| 15-HETE | ARA | down |
| 14(15)-DiHET | ARA | down |
| 12-HETE | ARA | down |
| 11(12)-DiHET | ARA | down |
| 8,9-EET | ARA | down |
| PGE1 | DGLA | down |
| 19(20)-DiHDPE(A) | DHA | down |
| 19(20)-EpDPE(A) | DHA | down |
| 16(17)-EpDPE | DHA | down |
| 13(14)-DiHDPE(A) | DHA | down |
| 20-HDHA | DHA | down |
| 14(S)-HDHA | DHA | down |
| 16-HDHA | DHA | down |
| 7(8)-DiHDPE(A) | DHA | down |
| 17-HDHA | DHA | down |
| 7,8-EpDPE | DHA | down |
| 11(12)-DiHETE | EPA | down |
| 14(15)-DiHETE | EPA | down |
| 14(15)-EpETE | EPA | down |
| 12(13)-DiHOME | LA | down |
| 9(S),12(S),13(S)-TriHOME | LA | down |

https://doi.org/10.3390/toxins18060260

| | | |
|---|---|---|
| 13(S)-HpODE | LA | down |
| 13(S)-HODE | LA | down |
| 12,13-EpOME | LA | down |
| 9(S),10(S),13(S)-TriHOME | LA | down |
| 9,10-DiHOME | LA | down |
| 9,10-EpOME | LA | down |
| 9-HODE | LA | down |

https://doi.org/10.3390/toxins18060260
